# Pelvic Pain and Adnexal Mass: Be Aware of Accessory and Cavitated Uterine Mass

**DOI:** 10.1155/2021/6649663

**Published:** 2021-02-11

**Authors:** Pooya Iranpour, Sara Haseli, Pedram Keshavarz, Amirreza Dehghanian, Neda Khalili

**Affiliations:** ^1^Medical Imaging Research Center, Shiraz University of Medical Sciences, Shiraz, Iran; ^2^Chronic Respiratory Diseases Research Center, National Research Institute of Tuberculosis and Lung Diseases (NRITLD), Shahid Beheshti University of Medical Sciences, Tehran, Iran; ^3^Department of Diagnostic & Interventional Radiology of New Hospitals LTD, Tbilisi, Georgia; ^4^Department of Pathology, Shiraz University of Medical Sciences, Shiraz, Iran; ^5^School of Medicine, Tehran University of Medical Sciences, Tehran, Iran

## Abstract

Accessory and cavitated uterine mass (ACUM) is a rare form of Mullerian anomaly that usually presents in young females with chronic cyclic pelvic pain and/or dysmenorrhea. This clinical entity is often underdiagnosed as it may be mistaken for other differential diagnoses, such as pedunculated myoma or adnexal lesions. Imaging modalities, including ultrasonography and magnetic resonance imaging (MRI), accompanied with relevant and suspicious clinical findings are important tools in making acorrect diagnosis. To date, surgical excision of the mass remains the mainstay of treatment,which provides significant symptom relief. In this study, we present a female adolescent with chronic pelvic pain since menarche who underwent laparotomy with the presumed diagnosis of a left-sided ovarian mass. Retrospective evaluation of pelvic MR images demonstrated that the lesion was in fact an ACUM, which was further confirmed by histopathological examination.

## 1. Introduction

Accessory and cavitated uterine mass (ACUM) is a rare newly described Mullerian anomaly, which generally presents with chronic recurrent pelvic pain and/or severe dysmenorrhea in women younger than 30 years of age [1]. It is an accessory mass at the insertion of the round ligament that is lined by normal endometrium and has a dark-brown-colored fluid content [2, 3]. ACUMs are difficult to diagnose because of their broad differential diagnosis, which includes rudimentary and cavitated uterine horns such as those found in other uterine malformations (e.g., bicornuate uterus), adenomyosis with cystic or degenerated areas, degenerated leiomyomas, and essential and primary dysmenorrhea [4]. To the best of our knowledge, fewer than 60 cases of ACUM are reported in the literature at the time of writing this paper. In this case report, we describe a 14-year-old girl presenting with severe pelvic pain since her menarche. Pelvic magnetic resonance imaging (MRI) showed an apparent adnexal mass, but final histopathological examination was in favor of ACUM.

## 2. Case Presentation

**Figure 1:**
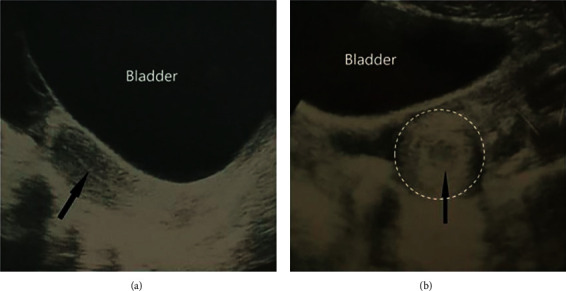
(a) On ultrasound, sagittal view of the pelvis shows the uterus with a normal uterine contour and normal echogenic endometrial line (arrow). (b) Axial view of the pelvis demonstrates a cross section of a mass-like lesion on the left side of the pelvic cavity, which was finally proved to be an accessory and cavitated uterine mass (arrow marks the endometrium).

**Figure 2:**
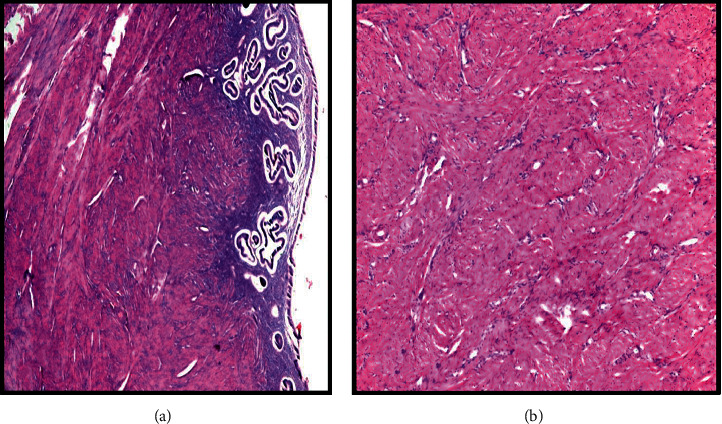
Histopathological examination of the pelvic mass shows (a) normal endometrial tissue in the innermost layer (hematoxylin and eosin, ×200) surrounded by (b) thick myometrial-type muscular layer (hematoxylin and eosin, ×200). Histopathological findings resembled a normal uterus, confirming the diagnosis of accessory and cavitated uterine mass.

**Figure 3:**
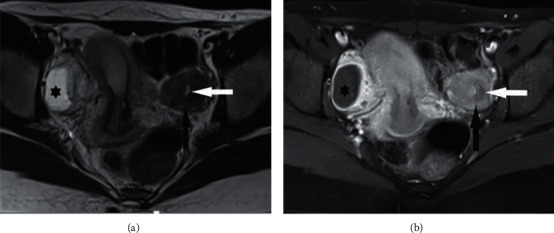
(a) Axial T2-weighted image of the pelvis shows a well-defined left-sided adnexal mass with signal intensity similar to the uterus (black arrow) with a central hypersignal endometrium (white arrow). (b) Axial contrast-enhanced fat-saturated T1-weighted image of the pelvis also demonstrates an enhancement similar to the uterus (black arrow). A right-sided simple ovarian cyst is also observed (asterisk). No pelvic lymphadenopathy is evident on magnetic resonance imaging.

**Figure 4:**
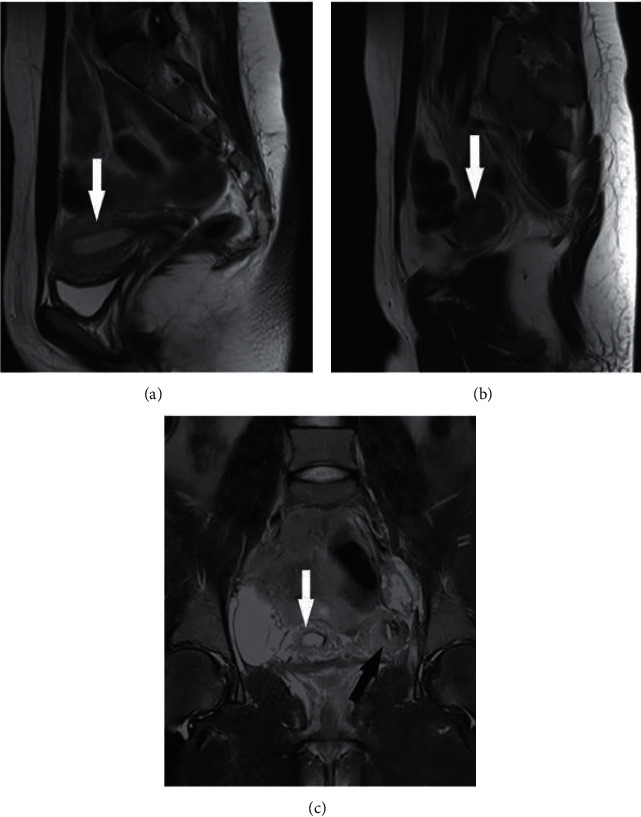
(a, b) Sagittal T2-weighted images of the pelvis reveal the uterus and the accessory and cavitated uterine mass, respectively. (c) Coronal T2-weighted image of the pelvis demonstrates the uterus on the right side (white arrow) and the accessory and cavitated uterine mass on the left side (black arrow), which are separate from each other.

A 14-year-old girl presented with chronic recurrent pelvic pain since her menarche. Her pain aggravated monthly during her menstruation; however, she had a regular and normal menstrual flow. Her personal history and family history were not significant, and she had no previous sexual history. Different kinds of anti-inflammatory medications had been prescribed for her during the past two years. The patient had also received oral contraceptive pills (OCPs) for two months, but none of the medications had been significantly effective. On physical examination, she was a well-developed girl with normal vital signs. She showed normal pubertal development with a height of 160 centimeters (percentile: 47%) and weight of 48 kilograms (percentile: 43%), and normal adrenarche and thelarche (stage 4). Pelvic examination of the external genitalia was normal with no evidence of imperforated hymen. No definite palpable mass was detected during abdominal examination. Ultrasonography was requested, which showed normal size and myometrial echogenicity of the uterus with normal endometrial thickness. There was evidence of a left-sided heterogeneous and mostly hyperechoic 35 × 30 mm adnexal mass with close contact to the left ovary (Figure 1). Color Doppler showed some internal vascularity. A small right-sided simple ovarian cyst was also seen on ultrasound. The cervix and vaginal canal were unremarkable on imaging. For further characterization of the mentioned lesion, pelvic MRI was performed, which revealed a well-defined heterogeneous mass with high signal intensity on T2-weighted images and low-to-intermediate signal intensity on T1-weighted images with significant enhancement after administration of gadolinium. The mass was located on the left side of the pelvic cavity, close to the left ovary and uterus; however, no connection to the uterus or ovary was detected. There was no pelvic lymphadenopathy or ascites. Based on the imaging findings, a paraovarian mass or a myoma was suspected. Subsequently, the patient underwent laparotomy, and a well-defined fleshy mass was seen on the left side of the pelvic cavity. The mass was located just under the round ligament, close to but separated from the left ovary with no adhesion to the surrounding structures. Both ovaries and uterus were normal except for a simple right-sided ovarian cyst with no endometrial implants. The uterus had two normally developed fallopian tubes with no fundal impression or contour abnormality and two normal cornua. On close observation, the uterus was completely separated from the mentioned mass. Several collateral vessels arising from the left uterine artery, which provided arterial supply to the mass, were ligated during surgery to obtain hemostasis. Frozen section revealed normal myometrial muscle with reactive endometrium. Finally, the mass was successfully resected by enucleation and incision through the serosa. While performing complete excision, a chocolate-colored fluid flowed out from the mass. The patient had an uneventful postoperative recovery and was discharged one day after surgery. She continued to be asymptomatic with complete resolution of the cyclic pelvic pain after one year of follow-up. Gross evaluation of the pelvic mass revealed a cavitated mass with a thick muscular wall, and pathological examination showed normal irregular myometrium lined by functional endometrium (Figure 2). Thus, a final diagnosis of accessory and cavitated uterine mass was made. Retrospective re-evaluation of pelvic MRI demonstrated a well-defined mass with signal intensity similar to the uterus on both T1-weighted and T2-weighted sequences with central hypersignal endometrium and normal endometrial-myometrial interface (Figures 3 and 4).

## 3. Discussion

Genitourinary development results from a complex interaction between the Wolffian ducts and the Mullerian ducts. Mullerian ducts are formed through invagination of the dorsal coelomic epithelium at about gestational week 6. At around 10 to 12 weeks of gestation, the two Mullerian ducts, fused with each other in a craniocaudal direction,finally fuse with the urogenital sinus, forming the uterus, bilateral fallopian tubes and the upper one-third of the vagina consequently [5, 6].

Mullerian anomalies are deviations from normal anatomy resulting from distortion of the complex embryogenesis. Although these anomalies are usually benign conditions, they may lead to significant reproductive problems based on the degree as well as the type of maldevelopment [6]. These commonly occurring anomalies can be found in about 4–7% of the population; so, an effective and reliable classification of Mullerian anomalies can contribute to proper diagnosis and management of affected individuals [7].

Mullerian anomalies were classified by Acien and colleagues in 1992 for the first time [8]; however, the European Society of Human Reproduction and Embryology and the European Society for Gynecological Endoscopy (ESHRE/ESGE) recently updated the classification system of female genital anomalies [7]. In this categorization, the main classes are designed on the basis of deviations from normal uterine anatomy deriving from the same embryological origin, while the clinical significance and degree of deformity are the basis for subclasses classification [7].

Uterine-like mass (ULM) is a distinct clinical entity defined as a cavitated mass lined with functional endometrium that is composed of sex hormone-sensitive smooth muscle cells, which are arranged irregularly at the periphery of the mass. These masses can be located within the uterus or anywhere else outside the uterus. ACUM, a subtype of non communicating ULM, is a rare newly described anomaly that, unlike other Mullerian anomalies, is associated with an otherwise normal uterus; hence, it should be classified as a separate entity [9]. Persistence or duplication of Mullerian duct due to a gubernaculum dysfunction at the site of round ligament attachment has been postulated as the pathogenesis of ACUM [9].

The same appearing masses have been described in the literature by different names, including cavitated adenomyoma, accessory cavitated mass, and juvenile cystic adenomyoma. However, ACUM is different from cystic adenomyosis, which usually occurs in middle-aged women, and is characterized by diffusely distributed adenomyotic foci within the uterus. Also, cystic adenomyosis lacks the normal endometrium and myometrium organization [10, 11].

The current criteria used for the diagnosis of ACUM includes: (1) an isolated accessory cavitated mass; (2) normal uterus (endometrial cavity), tubes, and ovaries; (3) surgical case with excised mass and with pathological examination; (4) accessory cavity lined by endometrial epithelium with glands and stroma; (5) chocolate-brown-colored fluid content; and (6) no adenomyosis (if uterus removed), but there could be small foci of adenomyosis in the myometrium adjacent to the accessory cavity [9]. The present case fulfilled all of the diagnostic criteria for ACUM; in addition, since the uterus, fallopian tubes, and ovaries were normal in this patient, other Mullerian anomalies were ruled out. ACUM has multiple challenging differential diagnoses, such as cavitated uterine horn with degenerated area or rudimentary uterine horn; however, imaging modalities such as hysterosalpingography, ultrasound, and especially MRI can aid in making a proper diagnosis [1, 12, 13]. Although most of the reported ACUM cases are located on the right lateral wall of the uterus near the round ligament, about one-third of cases are adjacent to the left round ligament, as in our case [14].

Table 1 presents a summary of the main findings of patients with ACUM that have been reported in previous studies. The most common symptoms in patients with ACUM are severe dysmenorrhea and recurrent pelvic pain that may occur before, during, or after menstruation [4]. Affected individuals usually experience severe cyclic pelvic pain and dysmenorrhea due to the presence of functional accessory endometrium [10].

Patients usually undergo surgical excision of the mass, and most of them have a presumed diagnosis of adnexal mass or pedunculated myoma preoperatively [11]. However, ACUM is diagnosed when an isolated cavitated mass is resected via surgery in patients with otherwise normal uterus, fallopian tubes, and ovaries [19].

Conclusively, ACUM is a rare Mullerian anomaly and treatable cause of severe dysmenorrhea in young females. To the best of our knowledge, most cases, as ours, are misdiagnosed before surgery, and diagnosis can only be confirmed after histopathological examination; hence, we recommend imaging modalities such as MRI for better evaluation. Pelvic MRI is highly accurate in making a diagnosis of ACUM in young women who present with early-onset dysmenorrhea and recurrent periodic pelvic pain, especially with otherwise normal uterus and ovaries. Although the true prevalence of ACUMs is not known yet, we believe that this clinical entity is in fact more common than currently thought. Thus, ACUM should be considered in the differential diagnosis of patients with clinically relevant symptoms.

## Figures and Tables

**Table 1:** Summary of previously reported cases of ACUM regarding their clinical features and management.

Reference	Age at diagnosis (years)	Symptoms	Preliminary diagnosis	Type of operation
Present study	(a) 14	(a) Chronic pelvic pain for two years	Para-ovarian mass or myoma	Laparotomy
Supermaniam et al. [15]	(a) 22 (b) 36	(a) A 3-month history of severe pain after menses (b) Chronic pelvic pain and severe dysmenorrhea since menarche	(a) Endometrioma or unicornuate uterus with a noncommunicating rudimentary horn (b) ACUM or degenerating fibroid	(a, b) Laparoscopy
Paul et al. [2]	(a) 19 (b) 17 (c) 25	(a) Chronic lower abdominal pain and severe dysmenorrhea for 4 years (b) Dysmenorrhea for 2 years (c) Dysmenorrhea for 7 years	(a) Uterine bicornis with a right horn hematometra (b) N/A (c) An obstructed rudimentary horn	(a, b, c) Laparoscopy
Bedaiwy et al. [16]	(a) 16	(a) Severe dysmenorrhea and cyclic pelvic pain since menarche	Rudimentary horn or cystic adenomyosis	Laparoscopy
Jain et al. [17]	(a) 19 (b) 22	(a) Severe dysmenorrhea and menorrhagia for the last three months (b) Severe dysmenorrhea, menorrhagia and secondary infertility for two years	(a) Uterus bicornis with a hematometra in obstructed rudimentary horn (b) Broad ligament fibroid	(a, b) Laparoscopy
Acien et al. [9]	(a) 15 (b) 21 (c) 33 (d) 32 (e) 48	(a) Severe, right hypogastric pain recurrent for more than one year (b) Severe dysmenorrhea and hypogastric pain for the past three years (c) Daily right iliac fossa pain during the previous few months (d) Intense dysmenorrhea, dyspareunia, right iliac fossa pain, and hypermenorrhea (e) Severe pain and hypermenorrhea	(a) Rudimentary uterine horn with an endometrial cavity containing thick endometrium (b) Cavitated adenomyoma (c) Subserous myoma (d) Endometrioma (e) Multiple myoma and cavitated adenomyoma	(a, b) Laparotomy (c) Laparoscopy (d) N/A (e) Total hysterectomy
Acien et al. [4]	(a) 36 (b) 20 (c) 18 (d) 19	(a) Abdominal pain (more intense in the left iliac fossae and increased during menstruation) (b) An 8-month history of left iliac fossae pain and progressive dysmenorrhea (c) Left iliac fossae pain, hypogastric pain, and progressive dysmenorrhea (d) Pelvic pain and progressive dysmenorrhea with increased pain following menstruation	(a) Degenerated leiomyoma or cystic adenomyoma (b) Accessory cavitated mass with the appearance of an endometrioma (c) Cystic adenomyosis (d) Myoma like structure	(a) Abdominal hysterectomy (b) Laparotomy with tumorectomy (c, d) Laparotomy
Na et al. [11]	(a) 39	(a) Continuous abdominal pain in the right lower quadrant (with radiation to the right thigh)	Ovarian endometriosis	Laparoscopy
Takeuchi et al. [18]	(a) 30 (b) 29 (c) 27 (d) 20 (e) 30 (f) 28 (g) 23 (h) 20 (i) 20	(a) Pelvic pain (b) Pelvic pain and dyspareunia (c) Dyspareunia (d) Pelvic pain (e) N/A (f) Dyspareunia (g) Pelvic pain (h) Pelvic pain (i) Pelvic pain	Cystic adenomyoma	(a–i) Laparoscopic tumor enucleation

N/A, not available; ACUM, accessory and cavitated uterine mass.

## Data Availability

The data and material used in this study will be available from the corresponding author upon request.

## Abbreviations:

ACUM: Accessory and cavitated uterine mass
MRI: Magnetic resonance imaging
OCP: Oral contraceptive pill
ULM: Uterine-like mass.
